# The Mediating Role of Organizational Commitment in the Relationship Between Perceived Organizational Climate and Quiet Quitting Among Nurses: A Cross-Sectional Study

**DOI:** 10.3390/healthcare14142123

**Published:** 2026-07-15

**Authors:** Esra Tansel Dalkın, Nihal Ünaldı Baydın

**Affiliations:** 1 Balaç Family Health Center, Samsun 55000, Turkey; esratanseltr@gmail.com; 2 Department of Nursing, Health Science Faculty, Ondokuz Mayıs University, Samsun 55000, Turkey

**Keywords:** nurses, organizational culture, organizational behaviour, work engagement, working conditions, quiet quitting

## Abstract

**Background/Objectives:** Perceived organizational climate has been associated with various work-related outcomes among nurses. Organizational commitment is expected to mediate the relationship between organizational climate and quiet quitting. This study aimed to investigate the mediating role of organizational commitment in the relationship between perceived organizational climate and quiet quitting among nurses. **Methods:** This cross-sectional, correlational, multicenter study was conducted with 453 nurses recruited through convenience sampling from four hospitals in Samsun, northern Türkiye. Data were collected between April and July 2025 using self-report instruments, including the Sociodemographic Information Form, Organizational Climate Scale, Organizational Commitment Scale, and Quiet Quitting Scale. Data were analyzed using IBM SPSS Statistics 26.0 and AMOS 24.0 through descriptive statistics, Pearson correlation analysis, and structural equation modeling. Statistical significance was set at *p* < 0.05. **Results:** The mean age of the nurses was 34.68 ± 8.99 years, and most participants were female (82.3%) and held a bachelor’s degree (83.9%). The mean quiet quitting score was 2.76 ± 0.77. Organizational climate had a significant direct effect on quiet quitting (β = −0.199; *p* < 0.001) and a positive effect on organizational commitment (β = 0.391; *p* < 0.001). Organizational commitment was negatively associated with quiet quitting (β = −0.267; *p* < 0.001). Organizational commitment partially mediated the relationship between organizational climate and quiet quitting (indirect effect: β = −0.104; *p* < 0.001). **Conclusions:** Organizational climate was associated with both organizational commitment and quiet quitting, with organizational commitment partially mediating this relationship.

## 1. Introduction

Healthcare systems have grown more complex in recent years due to population aging, the rising prevalence of chronic diseases, and increasing expectations for patient safety [1]. These transformations often increase nurses’ workload and the organizational pressures they face [2]. In such settings, factors such as work-related stress, ethical dilemmas, and limited organizational support can gradually lead to emotional and behavioral withdrawal among healthcare professionals [3,4]. This withdrawal often manifests as employees limiting their efforts to core job responsibilities and experiencing a reduced psychological attachment to the organization, a phenomenon consistent with quiet quitting. In high-stakes environments such as nursing, where tolerance for error is minimal, such withdrawal may disrupt care processes and compromise patient safety [5,6,7].

Quiet quitting, a concept that has gained increasing attention in recent years, refers to employees remaining in their jobs while limiting their efforts to the formal requirements of their roles and withdrawing from discretionary contributions to the organization [8,9]. This behavior reflects a subtle shift, characterized by reduced extra-role performance and a tendency to operate at minimal effort [10,11]. Although quiet quitting may overlap conceptually with burnout, work disengagement, and turnover intention, it represents a distinct construct characterized by limiting effort to formal job requirements rather than complete psychological withdrawal or an intention to leave the organization [12]. Unlike burnout, which is characterized primarily by emotional exhaustion and reduced personal accomplishment [13], quiet quitting reflects a behavioral reduction in discretionary work effort. Similarly, employees who engage in quiet quitting do not necessarily intend to leave their organization; instead, they continue fulfilling their formal job responsibilities while limiting extra-role contributions and organizational involvement [12,14]. This pattern has significant implications at both the individual and organizational levels. For individuals, it may be associated with decreased motivation and limited professional growth; for organizations, it can undermine overall performance and, in healthcare settings, compromise the quality of care [15]. Evidence suggests that such patterns of disengagement have become increasingly visible among nurses [16]. These concerns highlight the need for a better understanding of the organizational factors that contribute to quiet quitting.

In this context, organizational climate plays a central role in shaping employee behavior. Organizational climate is a multidimensional construct that reflects employees’ perceptions of their work environment and influences their attitudes and actions [17]. It includes elements such as leadership style, communication patterns, and organizational practices, all of which play critical roles in shaping job satisfaction, motivation, and performance [18,19]. Previous research has shown that a negative organizational climate is associated with burnout and lower levels of performance, whereas a positive organizational climate is associated with higher employee engagement and better performance [4,20,21,22,23]. Together, these findings point to organizational climate as a potential predictor of quiet quitting behavior. Organizational commitment, defined as employees’ emotional and cognitive attachment to their organization, represents another critical factor, particularly in healthcare settings where it is closely linked to the quality of care [24]. Diminished levels of commitment have been associated with adverse outcomes, including burnout, lower job satisfaction, and increased turnover intentions [25,26,27]. According to Meyer and Allen’s three-component model, organizational commitment consists of affective, normative, and continuance dimensions [28]. Notably, continuance commitment reflects employees’ tendency to remain with an organization due to perceived costs of leaving or a lack of alternatives, and conceptually aligns with the notion of quiet quitting. In such cases, individuals may remain physically present while becoming psychologically disengaged. These perspectives suggest that the relationship between organizational climate and quiet quitting may be indirect, operating through organizational commitment as a mediating mechanism.

To provide a theoretical grounding for these relationships, Social Exchange Theory, Conservation of Resources Theory, and the Job Demands–Resources Model together form a complementary framework. Social Exchange Theory argues that employees shape their attitudes and behaviors in accordance with the organizational support they perceive [29]. Conservation of Resources Theory emphasizes that individuals strive to obtain, maintain, and protect valued resources and that when these resources are threatened or depleted, withdrawal behaviors may emerge as a coping mechanism [30]. The Job Demands–Resources Model suggests that job resources foster organizational commitment and reduce adverse outcomes, while elevated job demands can strain psychological resources, thereby increasing withdrawal behaviors [31]. These theoretical perspectives suggest that organizational climate, as an important job resource, can strengthen organizational commitment, whereas unfavorable climate conditions may trigger quiet quitting.

Examining these relationships within the context of the Turkish healthcare system is particularly important. Public hospitals in Türkiye are often characterized by hierarchical organizational structures, high patient volumes, and demanding workloads, factors that may be related to nurses’ perceptions of organizational climate and organizational commitment [32,33]. Despite growing interest in quiet quitting, little is known about how organizational climate and organizational commitment jointly influence quiet quitting among nurses. Unlike previous studies, which have generally examined organizational climate, organizational commitment, or quiet quitting separately, the present study investigates these variables within a single mediation model among nurses in the Turkish healthcare context. This gap represents a significant area for further research, with implications for understanding employee behavior in healthcare and improving management practices. Drawing on Social Exchange Theory, Conservation of Resources Theory, and the Job Demands–Resources Model, a positive organizational climate may be viewed as an important organizational resource linked to stronger organizational commitment and lower levels of quiet quitting. Within this integrated framework, organizational climate functions as an organizational resource, organizational commitment develops through reciprocal exchange processes, and subsequently serves as a psychological resource that may be associated with lower withdrawal behaviors. Accordingly, organizational commitment was conceptualized as a potential mechanism linking organizational climate and quiet quitting.

Accordingly, this study aimed to examine the mediating role of organizational commitment in the relationship between perceived organizational climate and nurses’ quiet quitting behavior. By improving understanding of the relationships among organizational climate, organizational commitment, and quiet quitting, this study seeks to contribute to the literature and to inform healthcare management practices.

## 2. Materials and Methods

### 2.1. Study Design

A multicenter cross-sectional correlational study was conducted to examine the relationship between perceived organizational climate and quiet quitting among nurses and to test the mediating role of organizational commitment in this relationship. In the research model, quiet quitting behavior was defined as the dependent variable, perceived organizational climate as the independent variable, and organizational commitment as the mediating variable. The study was reported in accordance with the STROBE guidelines for observational research [34].

### 2.2. Setting and Sample

The study was conducted in four healthcare institutions in Samsun, a city in the Black Sea region of Türkiye: one university hospital, one training and research hospital, and two state hospitals. The study population comprised 2.487 nurses working in these institutions. Using a convenience sampling method, 500 nurses who met the inclusion criteria were approached and invited to participate in the study. Of these, 453 nurses voluntarily agreed to participate and provided complete data, yielding a response rate of 90.6%. Eligibility criteria included being a registered nurse, having at least one year of professional experience, and agreeing to participate voluntarily.

Based on calculations for a known population, the minimum required sample size was determined to be 332. Moreover, a power analysis conducted using G*Power 3.1, based on an effect size of r = 0.19 reported in a previous study, indicated a minimum sample size of 349 at a 95% confidence level, α = 0.05, and 95% statistical power [35]. Because no previous study examining the specific mediation model investigated in the present study was identified, the effect size was derived from the most conceptually relevant available study that included one of the key study variables and reported a relationship-based effect size. This approach was considered appropriate because SEM is fundamentally based on relationships among variables. To account for potential data loss, the target sample size was increased, and the study was completed with 453 participants. The achieved sample size was considered adequate for structural equation modeling based on methodological recommendations and the satisfactory fit of the measurement and structural models [36,37,38].

### 2.3. Data Collection Procedure

Data were collected between April and July 2025 using self-report questionnaires, after obtaining the necessary institutional approvals. Participation was voluntary, and written informed consent was obtained from all participants. The questionnaires were administered face-to-face and collected anonymously. A pilot study involving 12 nurses was conducted to assess the clarity and comprehensibility of the data collection instruments. The pilot study was intended solely to evaluate the clarity, comprehensibility, and feasibility of the questionnaires rather than their psychometric properties. As the purpose of the pilot study was limited to assessing the clarity and comprehensibility of the questionnaires rather than testing their reliability or validity, a small sample of nurses was considered adequate for identifying potential difficulties in understanding and completing the instruments. No major revisions were required, and these participants were excluded from the main study. The average time required to complete the questionnaires was 10–15 min.

### 2.4. Measurements

Data were collected using the Sociodemographic Information Form, the Organizational Climate Scale, the Organizational Commitment Scale, and the Quiet Quitting Scale. The psychometric properties of all scales were re-evaluated within the current sample.

Sociodemographic Information Form: Participants’ sociodemographic and occupational characteristics were assessed using a sociodemographic information form developed by the researchers in line with the relevant literature [39,40]. The form consists of 17 items covering variables such as age, gender, marital status, education level, professional experience, work experience within the institution and the unit, employment type, unit and position, working conditions, and job satisfaction.

The Organizational Climate Scale: The Organizational Climate Scale was developed by Çetinkaya and Güleç [41] to assess nurses’ perceptions of the organizational climate. The scale comprises 48 items across eight subdimensions: trust and cohesion (TC), reward and sanction (RS), autonomy and decision-making (AD), role clarity (RC), coordination and communication (CC), organizational ethics (OE), balanced workload (BW), and supportive environment (SE). Items are rated on a five-point Likert scale (1 = strongly disagree, 5 = strongly agree). In this study, instead of a total scale score, each sub-dimension was evaluated based on its own mean score and included as separate observed variables in the structural equation model. Higher scores on a sub-dimension indicate a more positive perception of that specific dimension of organizational climate. The construct validity of the scale was verified using CFA in the current study, with detailed fit indices provided in Supplementary File S1. In the original study, Cronbach’s alpha coefficients for the subdimensions ranged from 0.89 to 0.96; in the present study, they ranged from 0.71 to 0.93.

The Organizational Commitment Scale: The Organizational Commitment Scale was developed by Meyer, Allen, and Smith [28] to assess employees’ level of commitment to their organization and was adapted into Turkish by Dağlı, Elçiçek, and Han [42]. The scale comprises 18 items across three subdimensions: affective commitment, continuance commitment, and normative commitment. Items are rated on a five-point Likert scale (1 = strongly disagree, 5 = strongly agree). Higher scores indicate higher levels of organizational commitment. To ensure construct validity within our sample, CFA was performed, and the statistical results are presented in Supplementary File S1. The Cronbach’s alpha coefficient was reported as 0.88 in the Turkish adaptation study and was calculated as 0.83 in the current study. For the purposes of the present study, organizational commitment was modeled as an overall construct using the total scale score. This approach was adopted to examine the mediating role of overall organizational commitment in the relationship between organizational climate and quiet quitting.

The Quiet Quitting Scale: The Quiet Quitting Scale was developed by Anand et al. [43] to assess quiet quitting behavior and was adapted into Turkish by Tiryaki Şen et al. [2] in a sample of nurses. The scale is unidimensional and consists of 7 items. Responses are rated on a five-point Likert scale (1 = strongly disagree, 5 = strongly agree), with higher scores indicating higher levels of quiet quitting. Scale scores were calculated based on item means. The validity of the tool was examined via CFA, and the comprehensive model fit findings are documented in Supplementary File S1. The Cronbach’s alpha coefficient was reported as 0.89 in the Turkish adaptation study and was found to be 0.71 in the present study.

### 2.5. Ethical Considerations

Ethical approval for the study was obtained from the University Social and Human Sciences Research Ethics Committee (28 February 2025; Decision No: 2025-188). Additionally, the necessary institutional permissions were obtained from the relevant hospitals and the Provincial Health Directorate. Following the receipt of all ethical and institutional approvals, participant recruitment and data collection were conducted between April and July 2025. Written informed consent was obtained from all participants prior to data collection, and participation was voluntary. Permissions to use all measurement instruments were also obtained. All data were collected anonymously and used only for scientific purposes. The study was conducted in accordance with the principles of the Declaration of Helsinki.

### 2.6. Data Analysis

The data were analyzed using IBM SPSS Statistics 22.0 (IBM Corp., Armonk, NY, ABD), IBM SPSS AMOS (Version 24.0; IBM Corp., Armonk, NY, USA), and GPower version 3.1 [44]. Prior to data collection, the required sample size was determined through power analysis using GPower. The normality of the data distribution was assessed by examining skewness and kurtosis values. Negatively worded items were reverse-coded before analysis. Frequencies, percentages, means, and standard deviations were used to summarize participants’ sociodemographic characteristics and scale scores. The construct validity of the measurement instruments was evaluated using CFA, and internal consistency was assessed using Cronbach’s alpha coefficients. Relationships between variables were examined using Pearson correlation analysis. For the structural model, organizational climate was represented by its eight subdimensions, reflecting the multidimensional structure of the scale. Organizational commitment and quiet quitting were modeled using total scores to represent the overall constructs examined in the study. Structural Equation Modeling (SEM), using the maximum likelihood estimation method, was employed to test the research model. Mediating effects were assessed using the bootstrap method with 5000 resamples. Model fit was evaluated using the following indices: χ^2^/df, CFI, IFI, TLI, NFI, RMSEA, and SRMR. No missing data were identified in the dataset; therefore, all questionnaires were included in the analyses. Prior to the analyses, the assumptions of SEM were evaluated. Skewness values within ±2 and kurtosis values within ±10 were considered indicative of acceptable normality [45]. Multicollinearity was assessed using tolerance and variance inflation factor (VIF) values. Tolerance values greater than 0.10 and VIF values below 10 were considered acceptable [36]. Skewness values ranged from −1.026 to 2.147, and kurtosis values ranged from −1.377 to 4.814, indicating acceptable univariate normality. No influential outliers were identified (maximum Mahalanobis distance = 14.18; maximum Cook’s distance = 0.07). Multicollinearity diagnostics indicated no concerns (r = 0.358, tolerance = 0.872, VIF = 1.147, and CI values ranging from 12.415 to 15.883). Therefore, the assumptions required for the subsequent analyses were considered to be met. Detailed assumption-testing results are provided in the Supplementary File S1. The level of statistical significance was set at *p* < 0.05. To assess the potential influence of common method variance resulting from the use of self-report measures collected at a single time point, Harman’s single-factor test was conducted. Common method variance was considered a potential concern if a single factor accounted for more than 50% of the total variance.

## 3. Results

### 3.1. Descriptive Statistics

Of the nurses, 82.3% were female, with a mean age of 34.68 ± 8.99 years. Overall, 60.5% were married, 83.9% held a bachelor’s degree, 92.5% worked as ward nurses, 41.5% worked in specialized units, and 68.4% worked in a shift system. The mean professional experience was 12.22 ± 9.73 years, the mean tenure in the current organization was 7.89 ± 8.48 years, and the mean tenure in the current unit was 5.12 ± 5.76 years. Furthermore, 79.5% of the nurses reported being satisfied with their institution and 83.7% with their unit, whereas 80.6% reported being dissatisfied with their salary (Table 1).

Among the organizational climate subdimensions, relatively higher mean scores were observed for coordination and communication (3.56 ± 0.80), trust and cohesion (3.44 ± 0.75), and autonomy and decision-making (3.31 ± 0.75), whereas the balanced workload dimension had the lowest mean score (2.92 ± 0.85). The mean quiet quitting score was 2.76 ± 0.77, while the mean organizational commitment score was 2.93 ± 0.53 (Table 2).

Positive statistically significant relationships were found between organizational climate subdimensions and organizational commitment. These relationships were generally weak, although moderate associations were observed for trust, cohesion and role clarity (*p* < 0.05; *p* < 0.001). Negative and statistically significant relationships were identified between quiet quitting and the subdimensions of organizational climate. While most associations were weak, moderate relationships were observed for trust, cohesion and organizational commitment (*p* < 0.001) (Table 2).

### 3.2. Measurement Model Results (Confirmatory Factor Analysis)

Confirmatory factor analyses were conducted to evaluate the construct validity of the study instruments. The Organizational Climate Scale demonstrated acceptable model fit (χ^2^/df = 3.141, CFI = 0.863, TLI = 0.869, IFI = 0.875, RMSEA = 0.069), the Organizational Commitment Scale showed acceptable fit (χ^2^/df = 3.660, CFI = 0.874, TLI = 0.859, IFI = 0.875, RMSEA = 0.077), and the Quiet Quitting Scale demonstrated good fit (χ^2^/df = 2.122, CFI = 0.979, NFI = 0.961, IFI = 0.980, RMSEA = 0.050).

Standardized factor loadings were statistically significant for all retained items. The complete standardized loading matrix is provided in Supplementary File S1 Standardized factor loadings ranged from 0.099 to 0.993 for the Organizational Climate Scale, 0.176 to 0.879 for the Organizational Commitment Scale, and 0.176 to 0.802 for the Quiet Quitting Scale. Although some indicators demonstrated relatively low factor loadings, all items were retained to preserve the original validated structure of the scales. In addition, these indicators represented theoretically relevant aspects of the constructs and were therefore retained despite their lower loadings. Composite reliability (CR) values ranged from 0.66 to 0.91 across the Organizational Climate subdimensions and were 0.89 and 0.70 for the Organizational Commitment and Quiet Quitting Scales, respectively. Average variance extracted (AVE) values ranged from 0.36 to 0.55 across the Organizational Climate subdimensions and were 0.34 and 0.28 for the Organizational Commitment and Quiet Quitting Scales, respectively. Although some AVE values were below the recommended threshold of 0.50, convergent validity was considered acceptable because the corresponding CR values exceeded 0.60, as suggested by Fornell and Larcker [46]. Discriminant validity was generally supported according to the Fornell–Larcker criterion. Detailed measurement model results are presented in Supplementary File S1. Organizational Climate was represented by its eight subdimensions, while Organizational Commitment and Quiet Quitting were included in the model using their total scores. During CFA, modification indices were reviewed, and a limited number of correlated error terms between theoretically related items within the same subdimension were specified to improve model fit while preserving the original factor structure of the scales. No items were deleted from any of the scales.

Harman’s single-factor test showed that the first unrotated factor accounted for 20.88% of the total variance, indicating that common method variance was unlikely to have substantially influenced the findings.

### 3.3. Structural Model Results

SEM was conducted to test the research model. Prior to analysis, key assumptions were assessed, and the data met the required conditions regarding sample size, multivariate normality, outliers, and multicollinearity.

The model demonstrated an acceptable fit according to the following indices (χ^2^/sd = 3.269, CFI = 0.95, IFI = 0.95, NFI = 0.91, TLI = 0.92, RMSEA = 0.07, SRMR = 0.04). In the structural model, perceived organizational climate had a significant direct negative effect on quiet quitting behavior (β = −0.199, *p* < 0.001). In addition, perceived organizational climate had a significant direct positive effect on organizational commitment (β = 0.391, *p* < 0.001), while organizational commitment had a significant direct negative effect on quiet quitting behavior (β = −0.267, *p* < 0.001).

The mediation analysis, conducted using the bootstrap method, revealed that the indirect effect of perceived organizational climate on quiet quitting behavior through organizational commitment was statistically significant (β = −0.104, 95% CI [−0.151, −0.063]). After including the mediating variable in the model, the direct effect remained significant (β = −0.199, *p* < 0.001), indicating partial mediation (Table 3).

Furthermore, the total effect of perceived organizational climate on quiet quitting behavior was significant (β = −0.304, 95% CI [−0.393, −0.208]) (Table 3).

In terms of explained variance, perceived organizational climate explained 15.3% of the variance in organizational commitment (R^2^ = 0.153), whereas perceived organizational climate and organizational commitment together explained 15.4% of the variance in quiet quitting behavior (R^2^ = 0.154) (Table 3).

**Figure 1:**
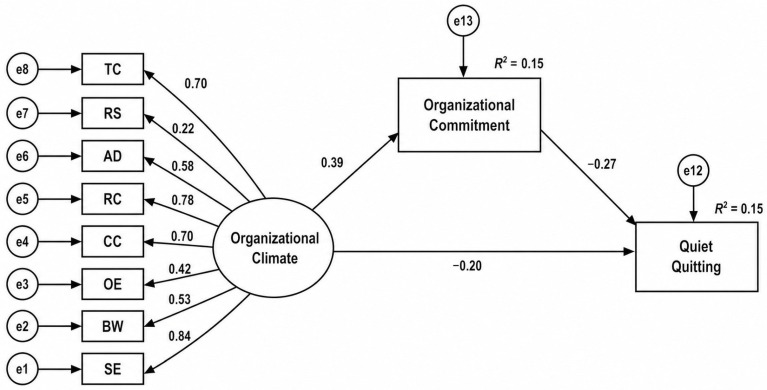
Structural equation model of the relationships among organizational climate, organizational commitment, and quiet quitting. Note: TC = trust and cohesion; RS = reward and sanction; AD = autonomy and decision-making; RC = role clarity; CC = coordination and communication; OE = organizational ethics; BW = balanced workload; SE = supportive environment; Standardized factor loadings and path coefficients are presented. Rectangles represent observed variables, ovals represent latent variables, and circles represent error terms. R^2^ values are reported for endogenous variables.

The fit indices for both the measurement and structural models are presented in Table 4, indicating acceptable to good model fit. The structural model with standardized path coefficients is illustrated in Figure 1, demonstrating the relationships among organizational climate, organizational commitment, and quiet quitting.

## 4. Discussion

This study examined the relationships among perceived organizational climate, organizational commitment, and quiet quitting among nurses and identified significant associations among these variables. The results suggest that organizational commitment mediated the relationship between organizational climate and quiet quitting, and that organizational climate was negatively associated with quiet quitting.

The relatively high scores observed both in the coordination and communication dimensions and the trust and cohesion dimensions suggest strong intra-team interaction and mutual trust in the work environment. However, the relatively low perceived balanced workload may indicate limitations in how workload distribution is experienced. This pattern suggests that, although nurses tend to evaluate interpersonal aspects of the work environment positively, structural challenges related to workload organization may persist. The relatively lower scores observed in the balanced workload dimension may reflect high patient volumes and intensive workload demands commonly reported in Turkish public hospitals [47,48]. In the present study, organizational climate was assessed through its multidimensional structure, whereas the literature has predominantly examined it using a total score approach. This subdimension-based assessment provides a more nuanced understanding of variation across different aspects of the work environment. Previous studies have generally reported nurses’ perceptions of organizational climate to be at or around moderate levels [49,50,51,52], although some findings indicate levels below moderate [23]. The current results suggest that nurses perceived coordination and trust more positively than participation in decision-making and equitable workload distribution.

The moderate level of organizational commitment observed among nurses is consistent with previous findings in the literature [53,54,55,56,57]. However, some studies have reported lower levels of organizational commitment [58], while others have found higher levels [59]. These variations may reflect contextual differences in organizational structures, management practices, and working conditions across institutions.

The moderate level of quiet quitting among nurses suggests that complete disengagement is not widespread, although there may be a decline in organizational participation and voluntary effort, consistent with studies reporting quiet quitting at moderate or low levels [6,14,60,61]. In contrast, some research has identified higher levels [1,62,63,64], which may be related to differences in working conditions and organizational stressors. Taken together, these findings suggest that quiet quitting may be associated not only with organizational factors and working conditions, but also with individual attitudes.

Perceived organizational climate was negatively associated with quiet quitting behavior. This finding is consistent with previous studies reporting that positive work environments are associated with lower levels of quiet quitting [65,66,67]. These findings suggest that lower levels of organizational withdrawal behavior may be observed in environments where employees feel valued, receive strong managerial support, and perceive work arrangements as fair. Some studies have reported positive associations between certain sub-dimensions of organizational climate and quiet quitting [68]. This discrepancy suggests that the relationships between organizational climate and quiet quitting may vary across its dimensions. In particular, environments characterized by uncertainty and insufficient supervision may be associated with greater reductions in discretionary work effort. However, the associations between quiet quitting and some organizational climate dimensions, particularly rewards and sanctions and organizational ethics, were relatively weak. This finding suggests that not all aspects of organizational climate contribute equally to quiet quitting behavior. While these dimensions may remain relevant to employees’ perceptions of the work environment, their practical influence on withdrawal-related behaviors may be more limited than that of communication, trust, support, and workload-related factors. Although rewards and sanctions and organizational ethics were less strongly associated with quiet quitting, they remain important dimensions of the organizational climate construct.

In addition, organizational climate was positively associated with organizational commitment. This finding is consistent with previous studies showing that a more positive organizational climate is associated with higher levels of employee commitment [20,21,69]. Supportive, trust-based, and collaborative work environments were associated with a stronger sense of belonging and organizational commitment among nurses. However, several of the statistically significant correlations observed in this study were weak. Given the relatively large sample size, statistically significant associations should not necessarily be interpreted as indicating strong practical effects. Therefore, these findings should be interpreted cautiously, with greater emphasis placed on the direction and consistency of the observed relationships than on their magnitude alone.

Organizational commitment was significantly and negatively associated with quiet quitting, indicating that higher levels of commitment corresponded to a lower tendency toward quiet quitting. This finding is consistent with previous studies reporting that supportive and positive work environments are associated with higher employee commitment and well-being and lower levels of quiet quitting [62,70,71]. Employees who feel emotionally connected to their organization and perceive themselves as integral members of it tend to report lower levels of withdrawal behaviors, suggesting that organizational commitment may be associated with both attitudes toward the organization and behavioral engagement at work. In addition, quiet quitting should not be viewed solely as a managerial problem. Previous research suggests that, although quiet quitting is associated with reduced organizational engagement, it may also represent an adaptive coping response to chronic occupational strain, allowing employees to limit excessive work demands and establish healthier boundaries between work and personal life [9]. Therefore, the present findings should be interpreted specifically within the context of quiet quitting behavior rather than broader employee well-being or turnover-related outcomes.

Organizational commitment was examined as an overall construct in the present study. Different dimensions of commitment may have distinct relationships with quiet quitting. In particular, continuance commitment may conceptually overlap with quiet quitting because both involve employees’ continued presence in the organization. However, these constructs are theoretically distinct. Continuance commitment reflects remaining in the organization because of the perceived costs of leaving or limited employment alternatives, whereas quiet quitting reflects a deliberate reduction in discretionary work effort while continuing to fulfill formal job responsibilities [12,28]. Therefore, potential differences among commitment dimensions could not be evaluated in the present study. Future studies should examine affective, continuance, and normative commitment separately to better understand their relationships with quiet quitting behavior.

The findings indicate that organizational commitment partially mediates the relationship between organizational climate and quiet quitting. A more positive perception of organizational climate was associated with greater organizational commitment, which in turn was associated with lower levels of quiet quitting. This finding is consistent with previous research highlighting the mediating role of organizational commitment in employee outcomes [72,73,74]. Similarly, research has shown that work environment conditions, workload, and leadership support are associated with organizational commitment and quiet quitting behavior [66,67,75,76]. These findings should be interpreted within the context of the Turkish healthcare system. Differences in organizational culture, workforce structures, management practices, and healthcare delivery systems across institutions and countries may influence organizational climate, organizational commitment, and quiet quitting, thereby limiting the transferability of the findings to other settings. However, the relatively modest proportion of variance explained for organizational commitment (R^2^ = 0.153) and quiet quitting (R^2^ = 0.154) suggests that these outcomes are influenced by a broader range of factors than those included in the present model. Therefore, organizational climate and organizational commitment should be viewed as contributing factors rather than as the sole or dominant determinants of quiet quitting behavior. The finding of partial rather than full mediation suggests that organizational climate may influence quiet quitting through additional mechanisms beyond organizational commitment. Other organizational, occupational, and individual factors not examined in the present study may also contribute to quiet quitting behavior. Therefore, the proposed model should be viewed as one possible explanation rather than a comprehensive account of all factors associated with quiet quitting.

The results suggest that quiet quitting may be associated with not only individual factors but also with organizational context, working conditions, and management practices. The findings are consistent with Social Exchange Theory and Conservation of Resources Theory, which propose that employees’ attitudes and behaviors are related to their perceptions of organizational support. In addition, the Job Demands–Resources Model provides a useful framework for understanding how job resources, such as communication, trust, and support, may be associated with organizational commitment and withdrawal behaviors [31]. Likewise, higher job demands, including excessive workload, may be associated with greater tendencies toward quiet quitting. Overall, a positive organizational climate was associated with lower levels of quiet quitting, both directly and indirectly through organizational commitment. These findings extend the current literature by highlighting organizational commitment as a potential mechanism linking organizational climate and quiet quitting. The study contributes to the literature by providing empirical evidence on the mediating role of organizational commitment, which remains relatively underexplored in research on quiet quitting.

These findings suggest that organizational-level strategies focusing on fair workload distribution, supportive leadership, open communication, and trust-based work environments may be relevant for addressing quiet quitting among nurses. Regular assessment of organizational climate and organizational commitment may also assist healthcare institutions in identifying early signs of quiet quitting and areas requiring organizational improvement.

### Limitations and Future Research

This study has several limitations. First, the study was conducted in only three public hospitals and one university hospital within a single province, which may limit the generalizability of the findings to nurses in other regions, private healthcare institutions, other countries, or different healthcare systems. Most participants were female staff nurses, which may further limit external validity. Differences in organizational culture, management practices, and workforce structures across healthcare settings may also affect how organizational climate, organizational commitment, and quiet quitting are experienced, and therefore should be considered when interpreting the findings. Second, the use of a convenience sampling method and the inclusion of only volunteer participants may reduce the representativeness of the sample and introduce selection bias. Third, all variables were measured using self-report questionnaires administered at a single time point, which may increase the risk of response bias, social desirability bias, and common method variance. To reduce this risk, participants completed the questionnaires anonymously and voluntarily. In addition, Harman’s single-factor test indicated that the first unrotated factor accounted for 20.88% of the total variance, suggesting that common method variance was unlikely to have substantially influenced the findings. Nevertheless, the limitations of this diagnostic approach should be acknowledged. Although the internal consistency of the Quiet Quitting Scale was acceptable (α = 0.71), it was lower than that of the other study instruments and should therefore be interpreted with caution. Further validation of the scale in different nursing populations is warranted [45,77]. Fourth, the cross-sectional design precluded establishing the temporal ordering of the variables; therefore, causal inferences cannot be drawn. Reverse or reciprocal relationships are also possible. For example, nurses with a higher degree of quiet quitting may perceive their organizational climate less favorably.

Fifth, because the primary aim of this study was to test the hypothesized mediation model, demographic and work-related characteristics were excluded as covariates from the SEM analysis. Nevertheless, the analyses did not account for all potential confounding variables, such as burnout, leadership style, psychological safety, staffing levels, or turnover intentions, which may have influenced the observed associations. Furthermore, participants were recruited from four healthcare institutions, and potential hospital-level clustering effects were not examined. Because of the small number of institutions, multilevel analyses were not performed; therefore, possible influence of clustering on parameter estimates and standard errors should be considered when interpreting the findings.

Finally, the model explained a relatively modest proportion of the variance in quiet quitting (R^2^ = 0.154), indicating that substantial variability in quiet quitting behavior remains unexplained. This finding suggests that quiet quitting is a multifactorial phenomenon influenced by a broader range of organizational, psychological, and contextual factors beyond those included in the present model. Future research should continue to examine additional determinants of quiet quitting and explore more comprehensive explanatory models.

## 5. Conclusions

This study examined the relationships among perceived organizational climate, organizational commitment, and quiet quitting among nurses and found that organizational commitment partially mediated the relationship between organizational climate and quiet quitting. The findings indicate that a more positive organizational climate was associated with higher organizational commitment and with lower levels of quiet quitting, both directly and indirectly through organizational commitment. However, the relatively modest proportion of variance explained suggests that additional organizational, psychological, and contextual factors also contribute to quiet quitting behavior. By improving understanding of the relationships among organizational climate, organizational commitment, and quiet quitting, this study contributes to the literature and may help inform healthcare management practices.

## Figures and Tables

**Table 1:** Nurses’ personal and professional characteristics (*n* = 453).

Characteristics	n	%	Min–Max	M ± SD
Gender				
Female	373	82.3		
Male	80	17.7		
Marital status				
Married	274	60.5		
Single	179	39.5		
Education level				
Highschool	42	9.3		
Bachelor’s	380	83.9		
Master and PhD	31	6.8		
Position				
Staff nurse	419	92.5		
Nurse manager	34	7.5		
Unit				
Medical	112	24.7		
Surgical	153	33.8		
Specialized unit (ER, ICU, etc.)	188	41.5		
Shift				
Day shift	131	28.9		
Night shift	12	2.6		
Rotating shifts	310	68.4		
Satisfied with the current organization				
Yes	360	79.5		
No	93	20.5		
Satisfied with the current unit				
Yes	379	83.7		
No	74	16.3		
Satisfied with salary				
Yes	88	19.4		
No	365	80.6		
Age			21–60	34.68 ± 8.99
Professional experience (years)			1–40	12.22 ± 9.73
Tenure in the current organization (years)			1–40	7.89 ± 8.48
Tenure in the current unit (years)			1–40	5.12 ± 5.76

Min–Max = minimum–maximum; M = mean; SD = standard deviation.

**Table 2:** Descriptive statistics, reliability (Cronbach’s alpha), and correlations of the scales and their subscales.

Variables	M ± SD	Min–Max	Cronbach’s Alpha	1	2	3	4	5	6	7	8	9	10
1. Coordination and Communication (CC)	3.56 ± 0.80	1.13–5.00	0.932	1									
2. Reward and Sanction (RS)	3.19 ± 0.76	1.00–5.00	0.741	0.162 ***	1								
3. Supportive Environment (SE)	3.16 ± 0.70	1.00–5.00	0.865	0.612 ***	0.348 ***	1							
4. Role Clarity (RC)	3.07 ± 0.84	1.00–5.00	0.792	0.497 ***	0.199 ***	0.675 ***	1						
5. Autonomy and Decision-making (AD)	3.31 ± 0.75	1.29–5.00	0.912	0.443 ***	0.086	0.503 ***	0.458 ***	1					
6. Trust and Cohesion (TC)	3.44 ± 0.75	1.00–5.00	0.798	0.541 ***	0.118 *	0.570 ***	0.409 ***	0.376 ***	1				
7. Organizational Ethics (OE)	3.02 ± 0.51	1.00–5.00	0.808	0.277 ***	0.155 ***	0.395 ***	0.320 ***	0.232 ***	0.264 ***	1			
8. Balanced Workload (BW)	2.92 ± 0.85	1.00–5.00	0.714	0.332 ***	0.131 **	0.407 ***	0.474 ***	0.326 ***	0.329 ***	0.362 ***	1		
9. Organizational Commitment (OC)	2.93 ± 0.53	1.11–4.44	0.834	0.262 ***	0.116 *	0.299 ***	0.314 ***	0.173 ***	0.362 ***	0.017	0.255 ***	1	
10. Quiet Quitting (QQ)	2.76 ± 0.77	1.00–5.00	0.712	−0.221 ***	−0.038	−0.225 ***	−0.218 ***	−0.065	−0.308 ***	−0.073	−0.239 ***	−0.345 ***	1

Notes: r = Pearson correlation coefficient; *p* < 0.001; M = mean; SD = standard deviation; Min–Max = minimum–maximum; CC = coordination and communication; RS = reward and sanction; SE = supportive environment; RC = role clarity; AD = autonomy and decision-making; TC = trust and cohesion; OE = organizational ethics; BW = balanced workload; OC = organizational commitment; QQ = quiet quitting; * *p* < 0.05; ** *p* < 0.01; *** *p* < 0.000.

**Table 3:** Mediation model results examining the role of organizational commitment in the relationship between organizational climate and quiet quitting.

	β_1_	β_2_	SE	CR	*p*	Effect Types
OCS → OC	0.355	0.391	0.044	8.069	<0.001	Direct
OCS → QQ	−0.260	−0.199	0.067	−3.902	<0.001	Direct
OC → QQ	−0.383	−0.267	0.068	−5.595	<0.001	Direct
OCS → OC → QQ 95% CI [−0.151, −0.063]	−0.136	−0.104	0.023	-	<0.001	Indirect
OCS → QQ (95% CI [−0.393, −0.208])	−0.396	−0.304	0.047	-	<0.001	Total effects

Notes: OCS = Organizational Climate Scale; QQ = Quiet Quitting Scale; OC = Organizational Commitment Scale; β_1_ = unstandardized coefficient; β_2_ = standardized coefficient; SE = standard error; CR = critical ratio; CI = confidence interval. Indirect and total effects were estimated using bootstrap with 95% confidence intervals. R^2^ values for endogenous variables were 0.153 for OC and 0.154 for QQ.

**Table 4:** Fit indices for the measurement and structural models.

Fit Index	Acceptable Value	Model 1	Model 2
χ^2^/df	<5	3.835	3.269
CFI	≥0.90	0.94	0.95
IFI	≥0.90	0.94	0.95
NFI	≥0.90	0.92	0.91
TLI	≥0.90	0.92	0.92
RMSEA	≤0.08	0.07	0.07
SRMR	≤0.08	0.04	0.04

Notes: χ^2^/df = chi-square divided by degrees of freedom; CFI = Comparative Fit Index; IFI = Incremental Fit Index; NFI = Normed Fit Index; TLI = Tucker–Lewis Index; RMSEA = Root Mean Square Error of Approximation; SRMR = Standardized Root Mean Square Residual.

## Data Availability

The data supporting the findings of this study are available from the corresponding author upon reasonable request. The data are not publicly available due to ethical and privacy restrictions.

## Supplementary Materials

The following supporting information can be downloaded at: https://www.mdpi.com/article/10.3390/healthcare14142123/s1, Supplementary File S1: Multivariate normality, multicollinearity, reliability analyses, KMO and Bartlett’s test results, and confirmatory factor analyses of the Organizational Climate Scale, Organizational Commitment Scale, and Quiet Quitting Scale and detailed measurement model evaluation results. Supplementary File S2: Permissions for scale use. Supplementary File S3: Ethics committee approval.

## Abbreviations

The following abbreviations are used in this manuscript:

CC	Coordination and Communication
RS	Reward and Sanction
SE	Supportive Environment
RC	Role Clarity
AD	Autonomy and Decision-making
TC	Trust and Cohesion
OE	Organizational Ethics
BW	Balanced Workload
OC	Organizational Commitment Scale
QQS	Quiet Quitting Scale
OCS	Organizational Climate Scale
CR	Critical Ratio
CFI	Comparative Fit Index
IFI	Incremental Fit Index
NFI	Normed Fit Index
TLI	Tucker–Lewis Index
RMSEA	Root Mean Square Error of Approximation
SRMR	Standardized Root Mean Square Residual
SEM	Structural Equation Modeling
CFA	Confirmatory Factor Analysis

## References

[1] Galanis P., Moisoglou I., Katsiroumpa A., Gallos P., Kalogeropoulou M., Meimeti E., Vraka I. (2025). Workload increases nurses’ quiet quitting, turnover intention, and job burnout: Evidence from Greece. AIMS Public Health.

[2] Tiryaki Şen H., Yurtsever D., Polat Ş. (2024). Validity and reliability study of ‘quiet quitting and quiet firing scales’ in Turkish: A study on nurses. J. Health Nurs. Manag..

[3] Sherman M., Klinenberg E. (2024). Beyond burnout: Moral suffering among healthcare workers in the first COVID-19 surge. Soc. Sci. Med..

[4] Toska A., Dimitriadou I., Togas C., Nikolopoulou E., Fradelos E.C., Papathanasiou I.V., Saridi M. (2025). Quiet quitting in the hospital context: Investigating conflicts, organizational support, and professional engagement in Greece. Nurs. Rep..

[5] Jun J., Ojemeni M.M., Kalamani R., Tong J., Crecelius M.L. (2021). Relationship between nurse burnout, patient and organizational outcomes: A systematic review. Int. J. Nurs. Stud..

[6] Kang J., Kim H., Cho O.H. (2023). Quiet quitting among healthcare professionals in hospital environments: A concept analysis and scoping review protocol. BMJ Open.

[7] Liu-Lastres B., Karatepe O.M., Okumus F. (2024). Combating quiet quitting: Implications for future research and practices for talent management. Int. J. Contemp. Hosp. Manag..

[8] Scheyett A. (2023). Quiet quitting. Soc. Work.

[9] Serenko A. (2024). The human capital management perspective on quiet quitting: Recommendations for employees, managers, and national policymakers. J. Knowl. Manag..

[10] Formica S., Sfodera F. (2022). The great resignation and quiet quitting paradigm shifts: An overview of current situation and future research directions. J. Hosp. Mark. Manag..

[11] Johar S.A., Hassan S.M., Saiyed H. (2023). Silent disengagement: Understanding the consequences of quiet quitting, trends, and impacts. Int. J. Clin. Stud. Med. Case Rep..

[12] Patel P.C., Guedes M.J., Bachrach D.G., Cho Y. (2025). A multidimensional quiet quitting scale: Development and test of a measure of quiet quitting. PLoS ONE.

[13] Maslach C., Leiter M.P. (2016). Understanding the burnout experience: Recent research and its implications for psychiatry. World Psychiatry.

[14] Kim K.T., Sohn Y.W. (2024). The impact of quiet quitting on turnover intentions in the era of digital transformation: The mediating roles of job satisfaction and affective commitment and the moderating role of psychological safety. Systems.

[15] Cho S.H., Lee J.Y., You S.J., Song K.J., Hong K.J. (2020). Nurse staffing, nurses’ prioritization, missed care, quality of nursing care, and nurse outcomes. Int. J. Nurs. Pract..

[16] Galanis P., Moisoglou I., Malliarou M., Papathanasiou I.V., Katsiroumpa A., Vraka I., Kaitelidou D. (2024). Quiet quitting among nurses increases their turnover intention: Evidence from Greece in the post-COVID-19 era. Healthcare.

[17] Obeng A.F., Zhu Y., Azinga S.A., Quansah P.E. (2021). Organizational climate and job performance: Investigating the mediating role of harmonious work passion and the moderating role of leader–member exchange and coaching. SAGE Open.

[18] Şakar Horuz S., Ünalan D. (2024). Hemşirelerde örgüt iklimi ile çalışan sağlığı ve güvenliği kültürü algısı arasındaki ilişki. J. Midwifery Health Sci..

[19] Kalhor R., Khosravizadeh O., Moosavi S., Heidari M., Habibi H. (2018). Role of organizational climate in job involvement: A way to develop the organizational commitment of nursing staff. J. Evid.-Based Integr. Med..

[20] Al-Motary M., Almowallad N., Al Hassan M., Alshmemri M., Alghabashi M.T. (2022). Impact of organizational climate and commitment among nurses job satisfaction: A review of literature. Asian J. Med. Health.

[21] Baş T., Amarat M., Ünal Ö., Durmuş A., Boz Ş. (2018). The effects of organizational climate on organizational commitment: The case of a private hospital. J. Mehmet Akif Ersoy Univ. Fac. Econ. Adm. Sci..

[22] Hassan M.A., Almowallad N., Motary M.A., Alshmemri M., Alghabbashi M. (2021). Impact of organizational climate on nurses’ commitment at public hospitals in Saudi Arabia. J. Pharm. Res. Int..

[23] Hossny E.K., Alotaibi H.S., Mahmoud A.M., Elcokany N.M., Seweid M.M., Aldhafeeri N.A., Abdelkader A.M., Elhamed S.M.A. (2023). Influence of nurses’ perception of organizational climate and toxic leadership behaviors on intent to stay: A descriptive comparative study. Int. J. Nurs. Stud. Adv..

[24] Stark H.P., Smith R.W., Carter N.T. (2025). Organizational commitment profiles and employee well-being: Exploratory and confirmatory latent profile analyses. Occup. Health Sci..

[25] Alotni M.A.A.S. (2019). The relationship between organisational commitment and burnout: A comparative study of nurses from a health care service. Am. J. Nurs. Res..

[26] Rodriguez-Fernandez M., Herrera J., de Las Heras-Rosas C., Ciruela-Lorenzo A.M. (2024). Practical implications of the organizational commitment model in healthcare: The case of nurses. J. Nurs. Manag..

[27] Suliman M., Aljezawi M. (2018). Nurses’ work environment: Indicators of satisfaction. J. Nurs. Manag..

[28] Meyer J.P., Allen N.J., Smith C.A. (1993). Commitment to organizations and occupations: Extension and test of a three-component conceptualization. J. Appl. Psychol..

[29] Blau P.M. (1964). Justice in social exchange. Sociol. Inq..

[30] Hobfoll S.E. (1989). Conservation of resources: A new attempt at conceptualizing stress. Am. Psychol..

[31] Bakker A.B., Demerouti E. (2007). The Job Demands–Resources model: State of the art. J. Manag. Psychol..

[32] Akkanat N.Z. (2025). Reform of the public hospitals union in the context of neoliberalism and health transformation policies. J. Int. Sci. Res..

[33] Berberoğlu A. (2018). Impact of organizational climate on organizational commitment and perceived organizational performance: Empirical evidence from public hospitals. BMC Health Serv. Res..

[34] von Elm E., Altman D.G., Egger M., Pocock S.J., Gøtzsche P.C., Vandenbroucke J.P. (2007). The Strengthening the Reporting of Observational Studies in Epidemiology (STROBE) Statement: Guidelines for Reporting Observational Studies. PLoS Med..

[35] Yıldız S., Katıtaş S., Doğan S. (2023). Always full performance! The relationship between burnout, organizational commitment and job performance in school administrators. Int. J. Lifelong Educ. Leadersh..

[36] Hair J.F., Risher J.J., Sarstedt M., Ringle C.M. (2019). When to use and how to report the results of PLS-SEM. Eur. Bus. Rev..

[37] Kline R.B., Brown G.G., Crosson B., Haaland K.Y., King T.Z. (2023). Structural equation modeling in neuropsychology research. APA Handbook of Neuropsychology: Neuroscience and Neuromethods.

[38] Yaslıoğlu M.M. (2017). Factor analysis and validity in social sciences: Application of exploratory and confirmatory factor analyses. Istanb. Univ. J. Sch. Bus..

[39] Göktepe N., Türkmen E., Fener İ., Yalçın B., Sarıköse S. (2021). Hemşirelerin bireysel, mesleki ve çalışma ortamı özelliklerinin bakım kalitesi algılarına etkisi. Sağlık Hemşire. Yönet. Derg..

[40] Ateş N., Erdal N., Harmancı Seren A.K. (2023). The relationship between critical thinking and job performance among nurses: A descriptive survey study. Int. J. Nurs. Pract..

[41] Çetinkaya A.Ş., Güleç G. (2023). İşletmelerde örgüt iklimi: Bir ölçek geliştirme çalışması. Marmara Üniversitesi İktisadi İdari Bilim. Derg..

[42] Dağlı A., Elçiçek Z., Han B. (2018). Adaptation of the “organizational commitment scale” into Turkish: Validity and reliability study. Electron. J. Soc. Sci..

[43] Anand A., Doll J., Ray P. (2024). Drowning in silence: A scale development and validation of quiet quitting and quiet firing. Int. J. Organ. Anal..

[44] Faul F., Erdfelder E., Lang A.-G., Buchner A. (2007). G*Power 3: A flexible statistical power analysis program for the social, behavioral, and biomedical sciences. Behav. Res. Methods..

[45] Collier J.E. (2020). Applied Structural Equation Modeling Using AMOS: Basic to Advanced Techniques.

[46] Fornell C., Larcker D.F. (1981). Evaluating Structural Equation Models with Unobservable Variables and Measurement Error. J. Mark. Res..

[47] Yıldırım N., Coskun H., Polat S. (2021). The relationship between psychological capital and the occupational psychologic risks of nurses: The mediation role of compassion satisfaction. J. Nurs. Scholarsh..

[48] Celebi Cakiroglu O., Tuncer Unver G. (2024). Toxic leadership, mental well-being and work engagement among nurses: A scale adaptation study and structural equation model approach. J. Health Organ. Manag..

[49] Almeida M., Barros V.G., da Silva S.M., da Silva F.J., Yamassake R.T., Telles A.C.M., Baptista P.C.P. (2023). Organizational climate, job satisfaction, and burnout in nursing workers. Rev. Bras. Med. Trab..

[50] Ersay Önal Z., Aba Y.A. (2023). The effects of organizational climate on organizational creativity perceptions of nurses. J. Health Nurs. Manag..

[51] Huo M., Qin H., Zhou X., Li J., Zhao B., Li Y. (2024). Impact of an organizational climate for evidence-based practice on evidence-based practice behaviour among nurses: Mediating effects of competence, work control, and intention for evidence-based practice implementation. J. Nurs. Manag..

[52] Ren Y., Song H., Li S., Xiao F. (2020). Mediating effects of nursing organizational climate on the relationships between empathy and burnout among clinical nurses. J. Adv. Nurs..

[53] Al-Haroon I., Al-Qahtani M.F. (2020). Assessment of organizational commitment among nurses in a major public hospital in Saudi Arabia. J. Multidiscip. Healthc..

[54] Das S., Latif A., Mallick D.R., Akter K. (2020). Organizational commitment perceived by clinical nurses in public hospitals of Bangladesh. J. Nurs. Health Sci..

[55] Gholami M., Saki M., Hossein Pour A.H. (2019). Nurses’ perception of empowerment and its relationship with organizational commitment and trust in teaching hospitals in Iran. J. Nurs. Manag..

[56] Labrague L.J., McEnroe-Petitte D.M., Tsaras K., Cruz J.P., Colet P.C., Gloe D.S. (2018). Organizational commitment and turnover intention among rural nurses in the Philippines: Implications for nursing management. Int. J. Nurs. Sci..

[57] Mon E.E., Akkadechanunt T., Chitpakdee B. (2022). Factors predicting organizational commitment of nurses in general hospitals: A descriptive-predictive study. Nurs. Health Sci..

[58] Kantek F., Yeşilbaş H. (2020). Investigation of nurses’ organizational commitment level: The case of Turkey. J. Int. Health Sci. Manag..

[59] Chang C.S. (2015). Moderating effects of nurses’ organizational support on the relationship between job satisfaction and organizational commitment. West. J. Nurs. Res..

[60] Gün I., Balsak H., Ayhan F. (2025). Mediating effect of job burnout on the relationship between organizational support and quiet quitting in nurses. J. Adv. Nurs..

[61] Rinaldi S., Pomarolli E. (2025). Quiet quitting among nurses: A case study in a Northern Italian hospital. Nurs. Rep..

[62] Domingue J.L., Lauzier K., Foth T. (2024). Quiet quitting: Obedience a minima as a form of nursing resistance. Nurs. Philos..

[63] Moisoglou I., Katsiroumpa A., Katsapi A., Konstantakopoulou O., Galanis P. (2025). Poor nurses’ work environment increases quiet quitting and reduces work engagement: A cross-sectional study in Greece. Nurs. Rep..

[64] Sezgin E.E. (2025). The relationship between quiet quitting and turnover intention in nurses: A systematic review and meta-analysis. Bus. Manag. Stud. Int. J..

[65] Çetiner N., Oğan E. (2025). The effect of climate on quiet quitting behavior: A study of academics. Istanb. Manag. J..

[66] Dai Z., Li J., Wang F., Wang L., Wang Y. (2023). Organizational management: Quiet quitting’s mitigation strategies for organizational response. J. Educ. Humanit. Soc. Sci..

[67] Xueyun Z., Al Mamun A., Masukujjaman M., Rahman M.K., Gao J., Yang Q. (2023). Modelling the significance of organizational conditions on quiet quitting intention among Gen Z workforce in an emerging economy. Sci. Rep..

[68] Gökçen Kapusuz A. (2025). Entropic organizational climate: An unseen antecedent of quiet quitting and social loafing behaviors. Cankiri Karatekin Univ. J. Inst. Soc. Sci..

[69] Bahrami M.A., Barati O., Ghoroghchian M.S., Montazer-Alfaraj R., Ranjbar Ezzatabadi M. (2016). Role of organizational climate in organizational commitment: The case of teaching hospitals. Osong Public Health Res. Perspect..

[70] Harris L.C. (2024). Commitment and quiet quitting: A qualitative longitudinal study. Hum. Resour. Manag..

[71] Pevec N. (2023). The concept of identifying factors of quiet quitting in organizations: An integrative literature review. Chall. Future.

[72] Cao Y., Liu J., Liu K., Yang M., Liu Y. (2019). The mediating role of organizational commitment between calling and work engagement of nurses: A cross-sectional study. Int. J. Nurs. Sci..

[73] Sharif Nia H., Arslan G., Naghavi N., Sivarajan Froelicher E., Kaveh O., Pahlevan Sharif S., Rahmatpour P. (2021). A model of nurses’ intention to care for patients with COVID-19: Mediating roles of job satisfaction and organisational commitment. J. Clin. Nurs..

[74] Xia G., Zhang Y., Dong L., Huang F., Pu Y., Luo J., Lei Z. (2023). The mediating role of organizational commitment between workplace bullying and turnover intention among clinical nurses in China: A cross-sectional study. BMC Nurs..

[75] Hungerford C., Jackson D., Cleary M. (2024). Quiet quitting, resenteeism and other forms of disengagement: What are the answers for nurses?. J. Adv. Nurs..

[76] Moisoglou I., Katsiroumpa A., Prasini I., Gallos P., Kalogeropoulou M., Galanis P. (2024). Innovation support reduces quiet quitting and improves innovative behavior and innovation outputs among nurses in Greece. Nurs. Rep..

[77] Galanis P., Katsiroumpa A., Vraka I., Siskou O., Konstantakopoulou O., Moisoglou I., Gallos P., Kaitelidou D. (2023). The Quiet Quitting Scale: Development and Initial Validation. AIMS Public Health.

