# Supplementary material for: The Mediating Role of Organizational Commitment in the Relationship Between Perceived Organizational Climate and Quiet Quitting Among Nurses: A Cross-Sectional Study
Source: Healthcare (Basel). 2026 Jul 15;14(14):2123. doi: 10.3390/healthcare14142123 (PMC13409879; doi:10.3390/healthcare14142123)
Supplement: Supplementary file 1 [file healthcare-14-02123-s001.zip › Supplementary File S1.pdf]

Supplementary File-S1

1. Results of Assumption Analyses

a) Multivariate Normality and Outliers

Table S1. Multivariate Normality Analysis of the Study Data

Scale	Skewness	Kurtosis
**Organizational Climate Scale (OCS)**		
OCS-1 Coordination and communication subscale	-0.632	-0.469
OCS -2 Coordination and communication subscale	-0.876	0.284
OCS -3 Coordination and communication subscale	-0.471	-0.482
OCS -4 Coordination and communication subscale	-0.567	-0.445
OCS -5 Coordination and communication subscale	-0.618	-0.055
OCS -6 Coordination and communication subscale	-0.556	-0.096
OCS -7 Coordination and communication subscale	-0.463	-0.068
OCS -8 Coordination and communication subscale	-0.480	-0.239
OCS -9 Reward and sanction subscale	0.209	-1.111
OCS -10 Reward and sanction subscale	-0.542	-0.119
OCS -11 Reward and sanction subscale	-0.527	-0.452
OCS -12 Reward and sanction subscale	-0.515	-0.366
OCS -13 Reward and sanction subscale	-0.355	-0.590
OCS -14 Supportive environment subscale	0.148	-1.083
OCS -15 Supportive environment subscale	-0.400	-0.666
OCS -20 Supportive environment subscale	-0.436	-0.469
OCS -21 Supportive environment subscale	-0.628	-0.154
OCS -45 Supportive environment subscale	-0.416	-0.500
OCS -48 Supportive environment subscale	-0.071	-0.561
OCS -16 Role clarity subscale	0.104	-0.640
OCS -17 Role clarity subscale	-0.365	-0.540
OCS -18 Role clarity subscale	-0.400	-0.242
OCS -19 Role clarity subscale	-0.060	-0.698
OCS -22 Autonomy and decision-making subscale	-0.401	-0.600
OCS -23 Autonomy and decision-making subscale	-0.236	-0.802
OCS -24 Autonomy and decision-making subscale	0.128	-0.773
OCS -25 Autonomy and decision-making subscale	-0.096	-0.733
OCS -26 Autonomy and decision-making subscale	-0.653	-0.142
OCS -27 Autonomy and decision-making subscale	-0.604	-0.267
OCS -28 Autonomy and decision-making subscale	-0.724	0.409
OCS -29 Trust and cohesion subscale	-0.692	-0.042
OCS -30 Trust and cohesion subscale	-0.550	-0.173
OCS -31 Trust and cohesion subscale	-0.649	0.214
OCS -32 Trust and cohesion subscale	-0.598	0.160
OCS -33 Trust and cohesion subscale	-0.536	0.143
OCS -34 Trust and cohesion subscale	-0.611	-0.159
OCS -35 Trust and cohesion subscale	-0.637	0.155
OCS -36 Trust and cohesion subscale	-0.464	-0.440
OCS -37 Trust and cohesion subscale	-0.645	-0.283
OCS -38 Organizational ethics subscale	-0.124	-0.868
OCS -39 Organizational ethics subscale	0.205	-0.393
OCS -40 Organizational ethics subscale	-0.044	-0.411
OCS -41 Organizational ethics subscale	-0.125	-0.557
OCS -42 Organizational ethics subscale	-0.454	-0.317
OCS -43 Balanced workload subscale	-0.439	-0.674

OCS -44 Balanced workload subscale	0.185	-0.694
OCS -46 Balanced workload subscale	-0.237	-0.514
OCS -47 Balanced workload subscale	-0.047	-0.762
Organizational Commitment (OC)		
OC - 1 Affective commitment subscale	-0.238	-0.518
OC - 2 Affective commitment subscale	-0.181	-0.820
OC -3 Affective commitment subscale	-0.023	-0.651
OC - 4 Affective commitment subscale	-0.180	-0.707
OC - 5 Affective commitment subscale	-0.174	-0.780
OC - 6 Affective commitment subscale	-0.324	-0.532
OC - 7 Continuance commitment	-1.026	1.013
OC - 8 Continuance commitment	-0.406	-0.448
OC - 9 Continuance commitment	0.063	-0.713
OC - 10 Continuance commitment	-0.276	-0.753
OC - 11 Continuance commitment	-0.078	-0.449
OC - 12 Continuance commitment	0.033	-0.670
OC - 13 Normative commitment	0.242	-0.737
OC - 14 Normative commitment	-0.258	-0.761
OC - 15 Normative commitment	0.590	-0.329
OC - 16 Normative commitment	-0.140	-0.653
OC - 17 Normative commitment	0.250	-0.778
OC - 18 Normative commitment	0.088	-0.678
Quiet Quitting Scale (QQS)		
QQS -1	2.147	4.814
QQS -2	-0.240	-1.377
QQS -3	1.226	0.905
QQS -4	-0.139	-0.953
QQS -5	-0.252	-0.791
QQS -6	0.178	-0.975
QQS -7	0.011	-1.122

b) Multicollinearity

Table S2. Multicollinearity Diagnostics for the Independent Variables

Variables	Multicollinearity Diagnostics				
	**Tolerance**	**VIF**	**CI**	**1**	**2**
1. Organizational Climate Scale	0.872	1.147	15.883	-	
2. Organizational Commitment Scale			12.415	0.358*	-

2. Results of Reliability

Reliability Analyses

Table S3. Reliability Analysis Results for the Study Variables

**Variables**	**Cronbach's Alpha Coefficient**
Organizational Climate Scale	0.931
Trust and Cohesion subscale	0.932
Reward and Sanction subscale	0.741
Autonomy and Decision-making subscale	0.865
Role Clarity subscale	0.792
Coordination and Communication subscale	0.912
Organizational Ethics subscale	0.798
Balanced Workload subscale	0.808
Supportive Environment subscale	0.714
**Organizational Commitment Scale**	0.834
Affective commitment subscale	0.765
Continuance commitment subscale	0.654
Normative commitment subscale	0.739
**Quiet Quitting Scale**	0.712

3. Validity Analysis

a)Kaiser–Meyer–Olkin (KMO) and Bartlett's Test of Sphericity

Table S4. KMO and Bartlett's Test Results for the Variables

**Variables**	KMO	X^2	*df*	*p*
Organizational Climate Scale	0.896	13932.054	1128	**< 0.001**
Organizational Commitment Scale	0.826	2641.402	153	**< 0.001**
Quiet Quitting Scale	0.731	614.643	21	**< 0.001**

b) Results of Confirmatory Factor Analysis

Table S5. Confirmatory Factor Analysis Results for The Organizational Climate Scale

Organizational Climate Scale	β_1	β_2	Std. Error	CR	p
OCS-1 Coordination and communication subscale	1.000	0.683			
OCS-2 Coordination and communication subscale	1.024	0.750	0.061	16.909	< 0.001
OCS-3 Coordination and communication subscale	1.012	0.726	0.073	13.825	< 0.001
OCS-4 Coordination and communication subscale	1.115	0.754	0.065	17.058	< 0.001
OCS-5 Coordination and communication subscale	1.018	0.799	0.068	15.024	< 0.001
OCS-6 Coordination and communication subscale	0.988	0.753	0.069	14.234	< 0.001
OCS-7 Coordination and communication subscale	0.979	0.804	0.065	15.075	< 0.001
OCS -8 Coordination and communication subscale	0.917	0.665	0.072	12.746	< 0.001
OCS-9 Reward and sanction subscale	1.000	0.183			
OCS-10 Reward and sanction subscale	1.750	0.429	0.479	3.656	< 0.001
OCS-11 Reward and sanction subscale	3.975	0.828	1.020	3.895	< 0.001
OCS-12 Reward and sanction subscale	4.712	0.993	1.206	3.907	< 0.001
OCS-13 Reward and sanction subscale	3.504	0.741	0.905	3.871	< 0.001
OCS-14 Supportive environment subscale	1.000	0.099			
OCS-15 Supportive environment subscale	3.033	0.329	1.500	2.022	0.043
OCS-20 Supportive environment subscale	8.528	0.991	4.057	2.102	0.036
OCS-21 Supportive environment subscale	6.015	0.710	2.875	2.092	0.036
OCS-45 Supportive environment subscale	8.551	0.986	4.068	2.102	0.036
OCS-48 Supportive environment subscale	2.654	0.323	1.312	2.023	0.043
OCS-16 Role clarity subscale	1.000	0.561			
OCS-17 Role clarity subscale	1.193	0.681	0.114	10.449	< 0.001
OCS-18 Role clarity subscale	1.081	0.660	0.105	10.293	< 0.001
OCS-19 Role clarity subscale	1.499	0.795	0.132	11.382	< 0.001
OCS-22 Autonomy and decision-making subscale	1.000	0.550			
OCS-23 Autonomy and decision-making subscale	1.335	0.664	0.111	12.003	< 0.001
OCS-24 Autonomy and decision-making subscale	1.571	0.766	0.142	11.069	< 0.001
OCS-25Autonomy and decision-making subscale	1.553	0.773	0.140	11.116	< 0.001
OCS-26 Autonomy and decision-making subscale	1.249	0.653	0.124	10.115	< 0.001
OCS-27 Autonomy and decision-making subscale	1.391	0.735	0.129	10.810	< 0.001
OCS-28 Autonomy and decision-making subscale	0.916	0.544	0.100	9.155	< 0.001
OCS-29 Trust and cohesion subscale	1.000	0.669			
OCS-30 Trust and cohesion subscale	1.300	0.817	0.084	15.532	< 0.001
OCS-31 Trust and cohesion subscale	1.146	0.773	0.077	14.946	< 0.001
OCS-32 Trust and cohesion subscale	1.191	0.838	0.073	16.223	< 0.001
OCS-33 Trust and cohesion subscale	1.167	0.835	0.073	15.981	< 0.001
OCS-34 Trust and cohesion subscale	0.899	0.599	0.075	11.924	< 0.001
OCS-35 Trust and cohesion subscale	0.912	0.615	0.075	12.200	< 0.001
OCS-36 Trust and cohesion subscale	1.074	0.676	0.081	13.339	< 0.001
OCS-37 Trust and cohesion subscale	0.871	0.544	0.080	10.874	< 0.001
OCS-38 Organizational ethics subscale	1.000	0.205			
OCS-39 Organizational ethics subscale	0.960	0.220	0.270	3.553	< 0.001
OCS-40 Organizational ethics subscale	4.360	0.927	1.042	4.182	< 0.001
OCS-41 Organizational ethics subscale	4.105	0.889	0.978	4.197	< 0.001
OCS-42 Organizational ethics subscale	1.081	0.253	0.327	3.309	< 0.001

OCS-43 Supportive environment subscale	1.000	0.606			
OCS-44 Supportive environment subscale	1.128	0.702	0.098	11.521	< 0.001
OCS-46 Supportive environment subscale	1.094	0.737	0.092	11.896	< 0.001
OCS-47 Supportive environment subscale	1.259	0.825	0.099	12.665	< 0.001

Model Fit Indices

**Fit Indices**	**Calculated Value**	**Acceptable Range**
x^2/df	3.141	< 5
CFI	0.863	> 0.85
TLI	0.869	> 0.85
IFI	0.875	> 0.85
RMSEA	0.069	< 0.08

*β1 = Unstandardized Path Coefficients; β2 = Standardized Path Coefficients

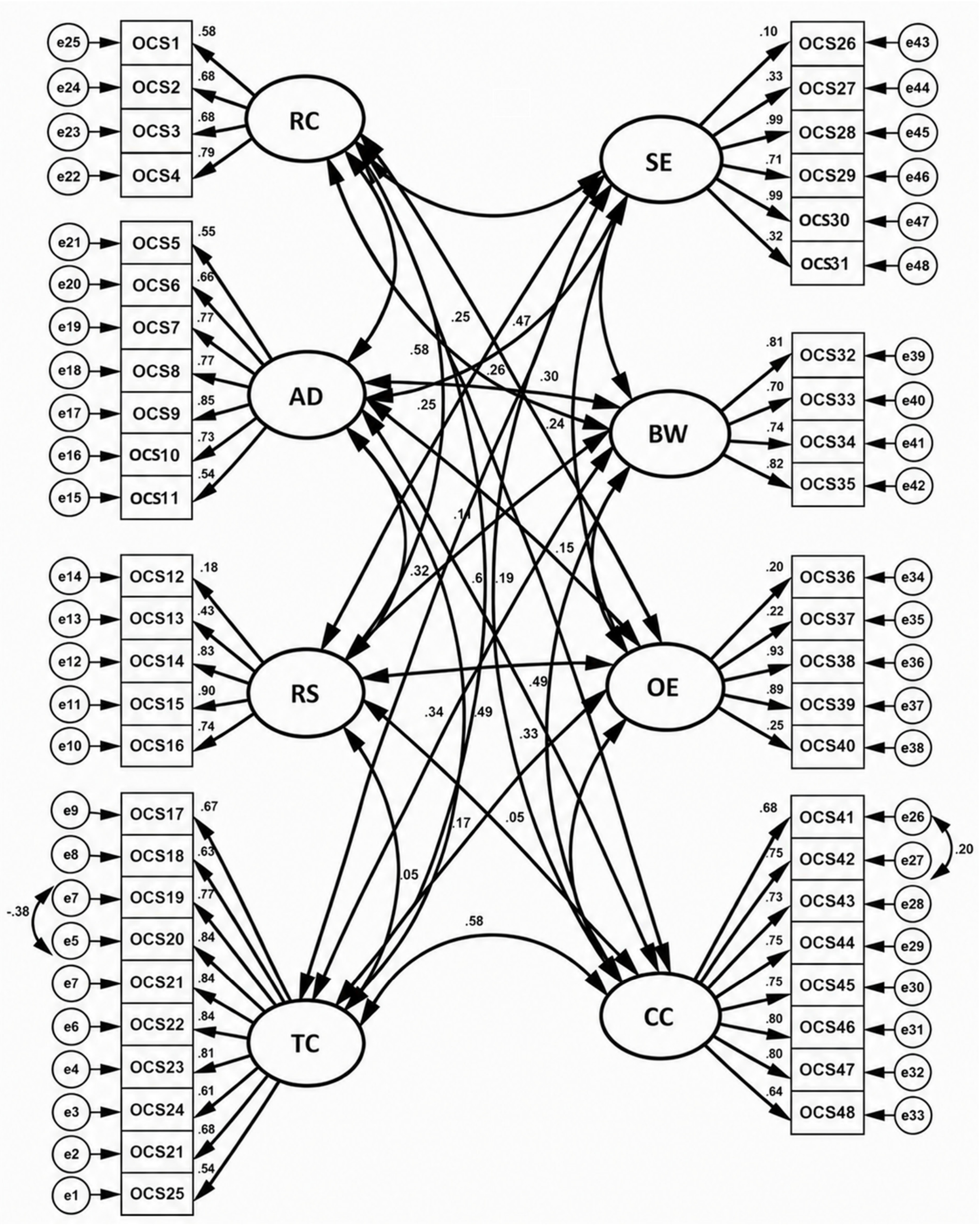

Figure S1. Model Structure of the Organizational Climate Scale

Notes: OCS= organizational climate; CC = coordination and communication; RS = reward and sanction; SE = supportive environment; RC = role clarity; AD = autonomy and decision-making; TC = trust and cohesion; OE = organizational ethics; BW = balanced workload

Confirmatory Factor Analysis of Organizational Commitment Scale

Table S6. Confirmatory Factor Analysis Results of the Organizational Commitment Scale

Organizational Commitment Scale	β_1	β_2	Std. Error	CR	p
OC 1 ← Affective commitment subscale	1.000	0.430			
OC 2 ← Affective commitment subscale	1.054	0.458	0.123	8.592	< 0.001
OC 3 ← Affective commitment subscale	1.549	0.679	0.187	8.305	< 0.001
OC 4 ← Affective commitment subscale	2.003	0.879	0.226	8.863	< 0.001
OC 5 ← Affective commitment subscale	1.654	0.728	0.194	8.526	< 0.001
OC 6 ← Affective commitment subscale	0.695	0.318	0.128	5.418	< 0.001
OC 7 ← Continuance commitment subscale	1.000	0.176			
OC 8 ← Continuance commitment subscale	2.737	0.455	0.829	3.300	< 0.001
OC 9 ← Continuance commitment subscale	3.790	0.582	1.203	3.150	0.002
OC 10 ← Continuance commitment subscale	3.624	0.563	1.154	3.141	0.002
OC 11 ← Continuance commitment subscale	2.939	0.498	0.947	3.104	0.002
OC 12 ← Continuance commitment subscale	3.302	0.553	1.053	3.136	0.002
OC 13← Normative commitment subscale	1.000	0.227			
OC 14← Normative commitment subscale	1.665	0.379	0.403	4.132	< 0.001
OC 15← Normative commitment subscale	2.569	0.609	0.572	4.493	< 0.001
OC 16← Normative commitment subscale	2.841	0.667	0.617	4.608	< 0.001
OC 17← Normative commitment subscale	3.487	0.779	0.745	4.683	< 0.001
OC 18← Normative commitment subscale	3.689	0.872	0.785	4.700	< 0.001

Model Fit Indices

**Fit Indices**	**Calculated Value**	**Acceptable Range**
x^2/df	3.660	< 5
CFI	0.874	> 0.85
TLI	0.859	> 0.85
IFI	0.875	> 0.85
RMSEA	0.077	< 0.08

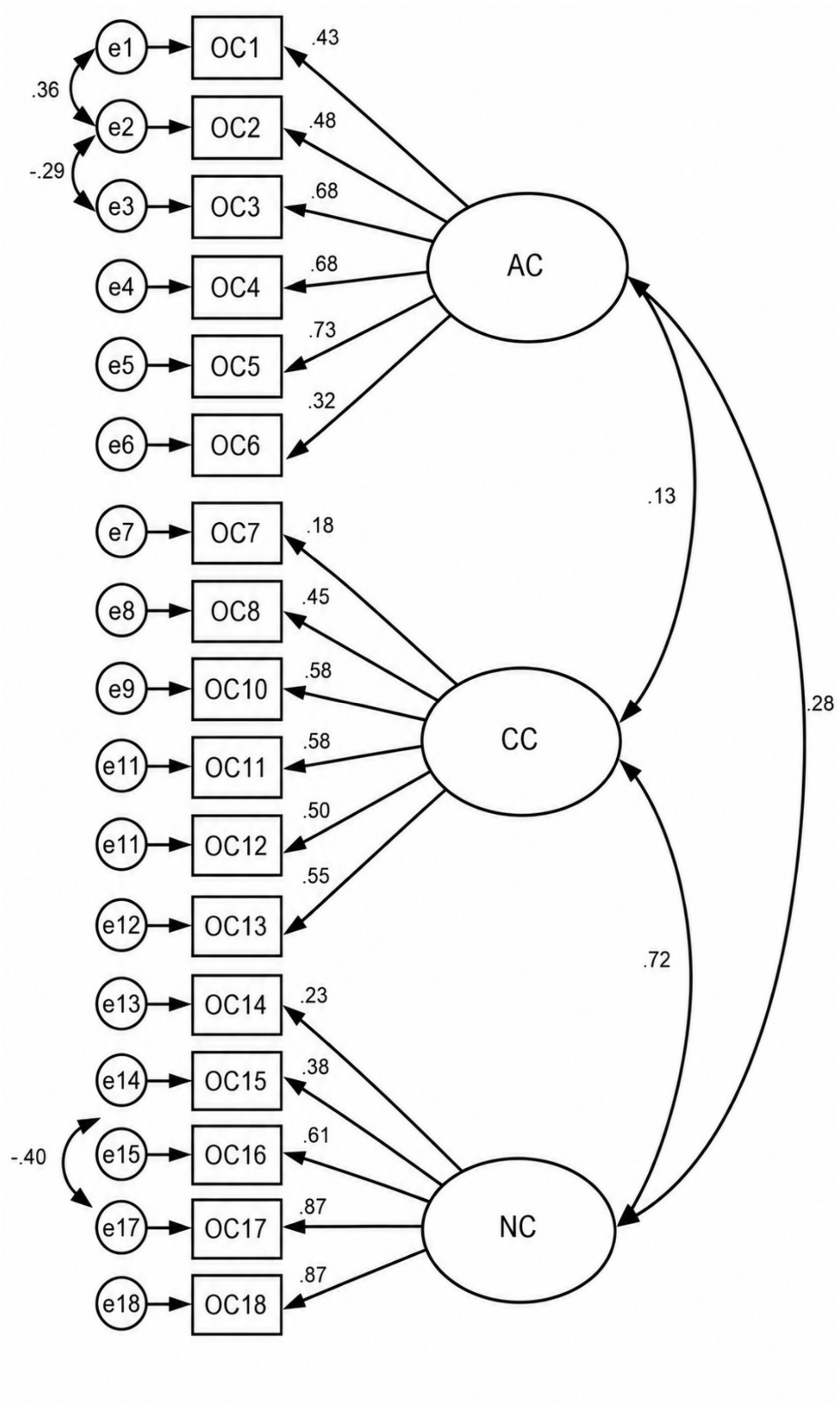

Figure S2. Model Structure of the Organizational Commitment Scale

Notes: OC = organizational commitment scale; AC= affective commitment; CC = continuance commitment; NC = normative commitment

Confirmatory Factor Analysis of Quiet Quitting Scale

Table S7. Confirmatory Factor Analysis Results of the Quiet Quitting Scale

**Quiet Quitting Scale**	**β_1**	**β_2**	**Std. Error**	**CR**	**p**
QQS1 ← Quiet Quitting Scale	1.000	0.176			
QQS 2← Quiet Quitting Scale	3.634	0.353	1.149	3.163	0.002
QQS 3← Quiet Quitting Scale	1.747	0.249	0.500	3.496	< 0.001
QQS 4← Quiet Quitting Scale	6.497	0.733	1.947	3.336	< 0.001
QQS 5← Quiet Quitting Scale	6.624	0.802	1.980	3.345	< 0.001
QQS 6← Quiet Quitting Scale	4.716	0.539	1.447	3.260	0.001
QQS 7← Quiet Quitting Scale	4.911	0.549	1.503	3.269	0.001

Model Fit Indices

**Fit Indices**	**Calculated Value**	**Acceptable Range**
x^2/df	2.122	< 5
CFI	0.979	> 0.95
NFI	0.961	> 0.95
IFI	0.980	> 0.95
RMSEA	0.050	< 0.95

*β1 = Unstandardized Path Coefficients; β2 = Standardized Path Coefficients

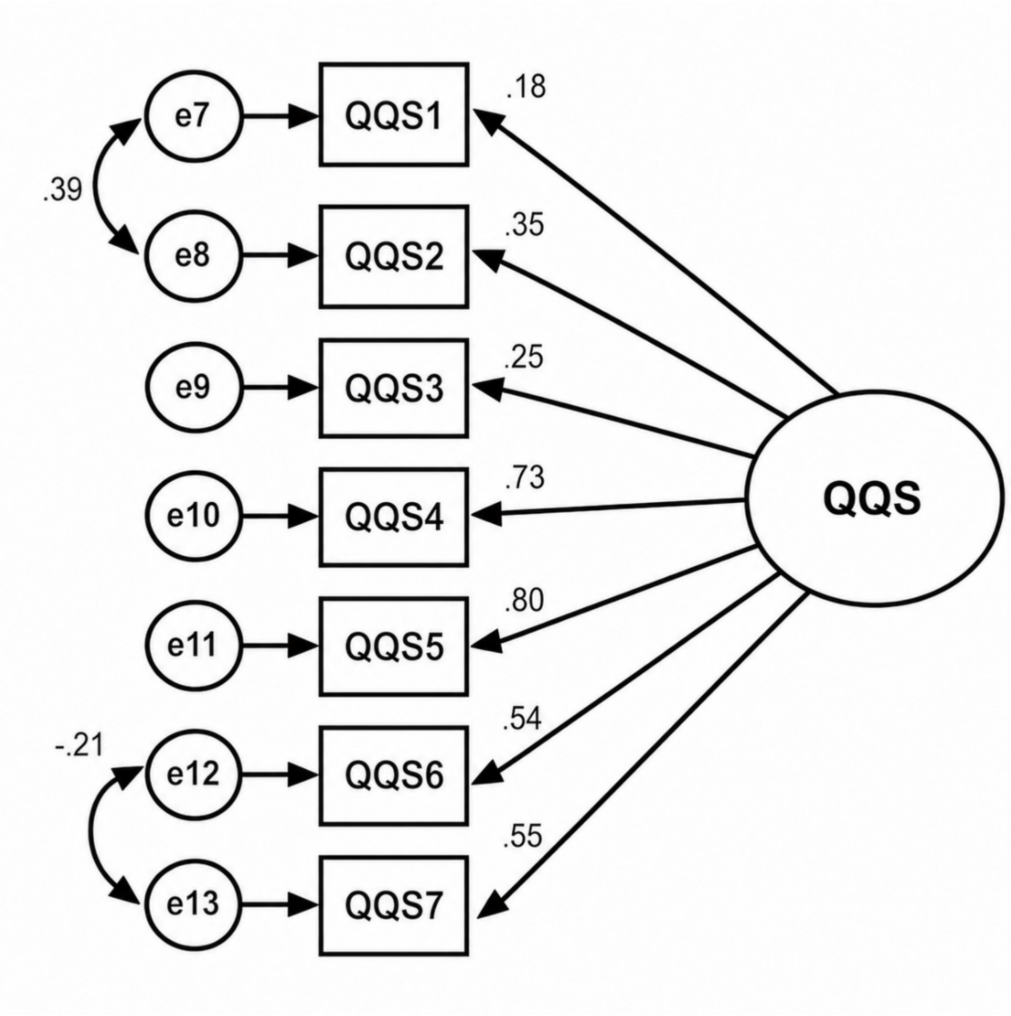

Figure S3. Model Structure of the Quiet Quitting Scale

Notes: QQS: Quiet Quitting Scale

4. Measurement Model Evaluation

Standardized Factor Loadings

Table S8 presents the standardized factor loadings of all indicators included in the measurement model. Standardized factor loadings ranged from 0.099 to 0.993 for the Organizational Climate Scale, from 0.176 to 0.879 for the Organizational Commitment Scale, and from 0.176 to 0.802 for the Quiet Quitting Scale. Although several indicators demonstrated relatively low factor loadings, all items were retained to preserve the original validated structure of the scales and because they represented theoretically relevant aspects of the constructs. The standardized measurement models are presented in Figure S1 (Organizational Climate Scale), Figure S2 (Organizational Commitment Scale), and Figure S3 (Quiet Quitting Scale).

Table S8. Standardized Factor Loadings of the Measurement Model

**Scale / Subdimension**	**Number of Items**	**Standardized Factor Loading Range (β)**
Organizational climate scale		
Coordination and communication	8	0.665–0.804
Reward and sanction	5	0.183–0.993
Supportive environment	6	0.099–0.991
Role clarity	4	0.561–0.795
Autonomy and Decision-making	7	0.544–0.773
Trust and Cohesion	9	0.544–0.838
Organizational ethics	5	0.205–0.927
Balanced workload	4	0.606–0.825
**Overall organizational climate scale**	48	0.099–0.993
**Organizational commitment scale**		
Affective commitment	6	0.318–0.879
Continuance commitment	6	0.176–0.582
Normative commitment	6	0.227–0.872
**Overall organizational commitment scale**	18	0.176–0.879
**Quiet quitting scale**	7	0.176–0.802

Note. Values represent standardized factor loadings obtained from confirmatory factor analyses. Detailed item-level factor loadings are provided in Table S5-S7.

Composite Reliability

The CR values of the scales included in the model were determined to be 0.98 for the Organizational Climate Scale and in the range of 0.66–0.91 for its subscales; 0.89 for the Organizational Commitment Scale as a whole; and 0.70 for the Quiet Quitting Scale as a whole. Based on the analyses, it was determined that composite reliability was established because the total CR values of the scales reached or exceeded the 0.70 threshold (Table S9).

Convergent Validity

The AVE values of the scales included in the model were determined to be 0.48 for the Organizational Climate Scale and in the range of 0.36–0.55 for its subscales; 0.34 for the Organizational Commitment Scale as a whole; and 0.28 for the Quiet Quitting Scale as a whole. As a result of the analyses, it was determined that composite reliability was established because the scales' total CR values reached or exceeded the 0.70 threshold. According to the literature, an AVE value greater than 0.50 is expected [1]. However, if the scales' CR values are higher than 0.6, AVE values below 0.5 are considered sufficient, and it is accepted that the scale has achieved convergent validity [2]. (Table S9)

Discriminant Validity

Discriminant validity was assessed using the Fornell–Larcker criterion. The Fornell–Larcker discriminant validity analysis was conducted only for the subscales of the Organizational Climate Scale, which are represented as subscales in the measurement model. Since the other scales were included in the structural model with their total scores (as observed variables), the Fornell–Larcker analysis was not applied to these constructs. According to the Fornell–Larcker criterion, the √AVE values for a subscale are expected to be greater than the correlations between that subscale and the other subscales. In this context, the square root of the AVE value for each sub-dimension (√AVE) was compared with the correlation coefficients between the sub-dimensions. Accordingly, the results showed that the √AVE values for the subscales Coordination and Communication, Rewards and Punishments, Task Clarity, Autonomy and Decision-Making, Trust and Harmony, Organizational Ethics, and Balanced Workload were higher than the correlation coefficients between these subscales and the other subscales. In the Supportive Environment subscale, however, the √AVE value (0.67) was found to be equivalent to the correlation coefficient with the Task Clarity subscale ($r = 0.67$). Nevertheless, the Fornell–Larcker criterion was met for all other subscale pairs, and the discriminant validity of the measurement model was assessed as generally acceptable (Table S10).

Table S9. Composite reliability (CR), average variance extracted (AVE), and square root of AVE (√AVE) values for the dimensions of the Organizational Climate Scale

**Subdimension**	**CR**	**AVE**	**√AVE**
Coordination and Communication	0.91	0.55	0.74
Reward and Sanction	0.80	0.49	0.70
Supportive Environment	0.78	0.45	0.67
Role Clarity	0.77	0.46	0.68
Autonomy and Decision-making	0.85	0.46	0.68
Trust and Cohesion	0.90	0.51	0.72
Organizational Ethics	0.66	0.36	0.60
Balanced Workload	0.81	0.53	0.72

Note. CR = Composite Reliability; AVE = Average Variance Extracted; √AVE = Square Root of the Average Variance Extracted

Table S10. Fornell–Larcker matrix for the dimensions of the Organizational Climate Scale

Subdimension	1	2	3	4	5	6	7	8
1.Coordination and Communication	**0.74**							
2. Reward and Sanction	0.16	**0.70**						
3. Supportive Environment	0.61	0.35	**0.67**					
4. Role Clarity	0.50	0.20	0.67	**0.68**				
5.Autonomy and Decision-making	0.44	0.09	0.50	0.46	**0.68**			
6. Trust and Cohesion	0.54	0.12	0.57	0.41	0.38	**0.72**		
7. Organizational Ethics	0.28	0.16	0.40	0.32	0.23	0.26	**0.60**	
8. Balanced Workload	0.33	0.13	0.41	0.47	0.33	0.33	0.37	**0.72**

Note. Bold diagonal values represent the square root of the average variance extracted (√AVE) for each subdimension, while the off-diagonal values represent the correlations between subdimensions.

REFERENCES

1. Yaslıoğlu, M.M. Factor analysis and validity in social sciences: Application of exploratory and confirmatory factor analyses. *Istanbul Univ. J. Sch. Bus.* **2017**, 46, 74–85
2. Fornell, C.; Larcker, D.F. Evaluating structural equation models with unobservable variables and measurement error. *J. Mark. Res.* **1981**, 18, 39–50. https://doi.org/10.2307/3151312
