# Supplementary material for: The Mediating Role of Organizational Commitment in the Relationship Between Perceived Organizational Climate and Quiet Quitting Among Nurses: A Cross-Sectional Study
Source: Healthcare (Basel). 2026 Jul 15;14(14):2123. doi: 10.3390/healthcare14142123 (PMC13409879; doi:10.3390/healthcare14142123)
Supplement: Supplementary file 1 [file healthcare-14-02123-s001.zip › Supplementary File S2-Permission to Use the Scales.pdf]

Permission to Use the Organizational Climate Scale

16.01.2025 16:45

Gmail Postalarda arayın

Ölçek izni kullanımı Gelen Kutusu ×

esra tansel
Alıcı: alisukru

Sayın hocam merhaba
Ben Ondokuz Mayıs Üniversitesi Lisansüstü Eğitim Enstitüsü Hemşirelik Yönetimi Anabilim Dalında yüksek lisans öğrencisiyim. Danışmanım yürüteceğim yüksek lisans tez çalışmamda kullanmak üzere geliştirdiğiniz Örgüt İklimi ölçeğinizden izniniz olursa yararlanmak istiyorum.
Saygılarımla.
Ondokuz Mayıs Üniversitesi Yüksek Lisans Öğrencisi
Esra TANSEL DALKIN

Ali Şükrü Çetinkaya
Alıcı: Gürkan, ben

Merhaba Esra Hanım,
Çalışmamıza olan ilginize teşekkür ederim. Ölçeği kullanabilirsiniz.
Çalışmanızda başarılar dilerim.
Selamlar,
Prof. Dr. Ali Şükrü Çetinkaya

Permission to Use the Organizational Commitment Scale

16.01.2025 16:47

Gmail

Postalarda arayın

ölçek kullanım izni

Gelen Kutusu ×

esra tansel
Alıcı: dagli

Sayın hocam merhaba
Ben Ondokuz Mayıs Üniversitesi Lisansüstü Eğitim Enstitüsü Hemşirelik Yönetimi Anabilim Dalında yüksek lisans öğrencisiyim. Danışmanım
yürüteceğim yüksek lisans tez çalışmamda kullanmak üzere geliştirdiğiniz "Örgütsel Bağlılık" ölçeğinizden izniniz olursa yararlanmak istiyoru
Saygılarımla.

Abidin DAĞLI
Alıcı: ben

Sn. Esra TANSEL,
Tarafımızdan Türkçeye uyarlanan *"Örgütsel Bağlılık Ölçeği"*ni çalışmalarınızda memnuniyetle kullanabilirsiniz. İyi çalışmalar diliyorum.
Sevgilerimle.

Prof. Dr. Abidin DAĞLI

Permission to Use the Quiet Quitting Scale

16.01.2025 16:48

Gmail in:sent

Ölçek kullanım izni

esra tansel
Alıcı: hanifetiryaki@gmail.com

Sayın hocam merhaba
Ben Ondokuz Mayıs Üniversitesi Lisanüstü Eğitim Enstitüsü Hemşirelik Yönetimi Anabilim Dalında yüksek lisans öğrencisiyim. Danışmanım Doç. Dr. Nihal Ünaldı E
çalışmamda kullanmak üzere Türkçeye uyarladığınız Sessiz İstifa ölçeğini izniniz olursa atıf yapmak koşuluyla kullanmak istiyoruz. Hocam Sessiz İstifa ölçeğini ayr
Sessiz İstifa ölçeğinin maddelerini paylaşırsanız çok seviniriz.
Saygılarımla.
Ondokuz Mayıs Üniversitesi Yüksek Lisans Öğrencisi
Esra TANSEL DALKIN

Yanıtla | Yönlendir

16.01.2025 16:47

Gmail Postalarda arayın

Sessiz istifa ve sessiz işten çıkarma ölçeği kullanım izni

Gelen Kutusu ×

Hanife Tiryaki Şen
Alıcı: ben

Merhaba Esra Hanım,
Ölçeği tabiki kulanabilirsiniz. Ölçek maddeleri ve ölçeğin makalesi ektedir. Kolaylıklar diliyorum.

Hanife TİRYAKİ ŞEN

2 ek • Gmail tarafından tarandı

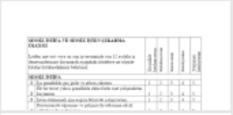

Sessiz İstifa ve Se.

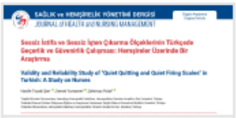

Sessiz istifa ve se.

Yanıtla | Yönlendir
