# Supplementary material for: The Mediating Role of Organizational Commitment in the Relationship Between Perceived Organizational Climate and Quiet Quitting Among Nurses: A Cross-Sectional Study
Source: Healthcare (Basel). 2026 Jul 15;14(14):2123. doi: 10.3390/healthcare14142123 (PMC13409879; doi:10.3390/healthcare14142123)
Supplement: Supplementary file 1 [file healthcare-14-02123-s001.zip › Supplementary File S3-Ethical Approval.pdf]

ONDOKUZ MAYIS ÜNİVERSİTESİ
SOSYAL VE BEŞERİ BİLİMLER ARAŞTIRMALARI ETİK KURUL KARARLARI

KARAR TARİHİ	TOPLANTI SAYISI	KARAR SAYISI
**28.02.2025**	**2**	**2025-188**

KARAR NO: 2025-188

Üniversitemiz Lisansüstü Eğitim Enstitüsü öğrencisi Esra TANSEL DALKIN'ın Doç. Dr. Nihal ÜNALDI BAYDIN danışmanlığında "Hemşirelerde Algılanan Örgüt İklimi, Örgütsel Bağlılık ve Sessiz İstifa Arasındaki İlişki" isimli yüksek lisans tezine ilişkin anket ve ölçek çalışmalarını içeren 41882 sayılı dilekçesi okunarak görüşüldü.

Üniversitemiz Lisansüstü Eğitim Enstitüsü öğrencisi Esra TANSEL DALKIN'ın Doç. Dr. Nihal ÜNALDI BAYDIN danışmanlığında "Hemşirelerde Algılanan Örgüt İklimi, Örgütsel Bağlılık ve Sessiz İstifa Arasındaki İlişki" isimli yüksek lisans tezine ilişkin anket ve ölçek çalışmalarının kabulüne oy birliği ile karar verildi.
